# Oral Pyronaridine Tetraphosphate Reduces Tissue Presence of Parasites in a Mouse Model of Chagas Disease

**DOI:** 10.1021/acsomega.4c05060

**Published:** 2024-08-20

**Authors:** Jair Lage Siqueira-Neto, Thomas R. Lane, Jean A. Bernatchez, Claudia Magalhaes Calvet Alvarez, Elany Barbosa da Silva, Miriam A. Giardini, Sean Ekins

**Affiliations:** † Center for Discovery and Innovation in Parasitic Diseases, Skaggs School of Pharmacy and Pharmaceutical Sciences, University of California, San Diego, La Jolla, California 92093, United States; ‡ Collaborations Pharmaceuticals, Inc., 840 Main Campus Drive, Lab 3510, Raleigh, North Carolina 27606, United States; § Laboratório de Ultraestrutura Celular, Instituto Oswaldo Cruz, FIOCRUZ, Rio de Janeiro, Rio de Janeiro 21040-300, Brazil

## Abstract

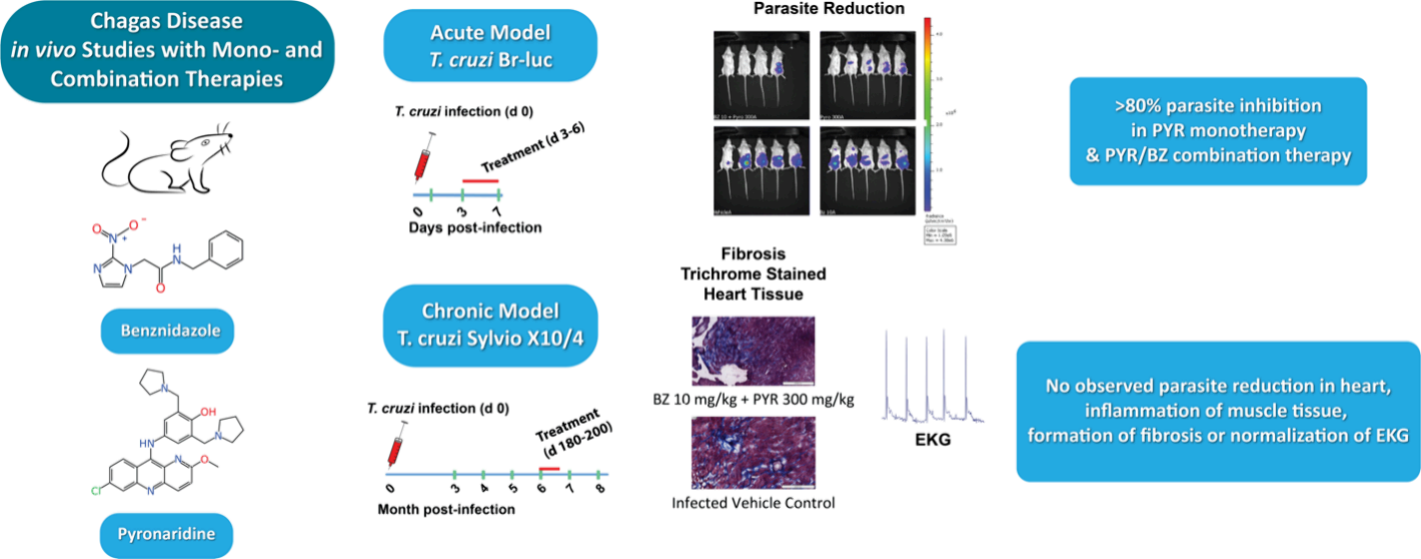

The eukaryotic parasite *Trypanosoma cruzi* (*T. cruzi*) is responsible for Chagas disease, which results in heart failure in patients. The disease is more common in Latin America, and is an emerging infection with The Centers for Disease Control estimating that greater than 300,000 people are currently infected in the United States. This disease has also spread from South and Central America, where it is endemic to many other countries, including Australia, Japan, and Spain. Current therapy for Chagas disease is inadequate due to limited efficacy in the indeterminate and chronic phases of the disease, in addition to the adverse effects from nifurtimox and benznidazole, which are nitro-containing drugs used for therapy. There is a clear need for new therapies for the Chagas disease. Using a computational machine learning approach, we have previously shown that the antimalarial pyronaridine tetraphosphate is active against *T. cruzi* Brazil-luc *in vitro* against parasites infecting a myoblast cell line and is also active *in vivo* in an acute mouse model of Chagas disease when dosed i.p. We now further evaluated oral pyronaridine as a monotherapy to determine the minimum effective dose to treat acute and chronic models of Chagas disease. Our results for *T. cruzi* Brazil-luc demonstrated daily oral dosing with pyronaridine from 150 to 600 mg/kg resulted in statistically significant inhibition in the 7 day acute mouse model. Combination therapy with daily dosing of benznidazole and pyronaridine in the acute infection model demonstrated that 300 mg/kg pyronaridine could return statistically significant antiparasitic activity to a subtherapetic 10 mg/kg benznidazole. In contrast, pyronaridine as monotherapy or combined with benznidazole lacked efficacy in the chronic mouse model, whereas 100 mg/kg benznidazole alone demonstrated undetectable parasites in the heart of mice. Pyronaridine requires further assessment in other chronic models to identify if it can be used beyond the acute stage of *T. cruzi* infection.

## Introduction

**Figure 1:**
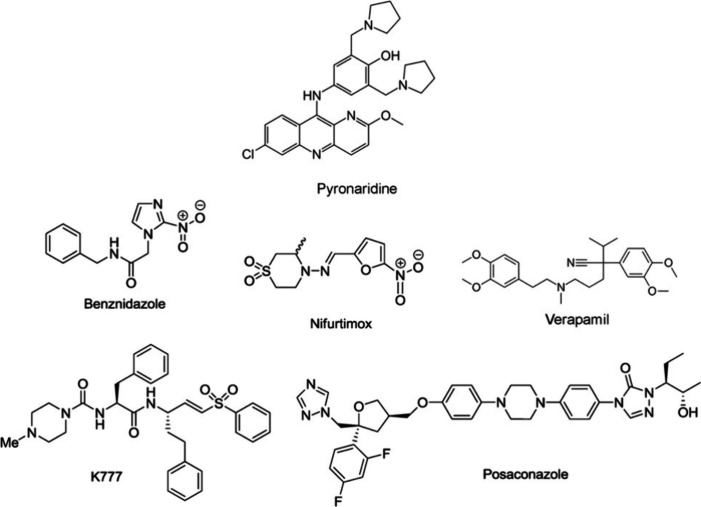
Pyronaridine^47^ and several other drugs^22−28^ in clinical use (benznidazole and nifurtimox) and others that are in development to treat Chagas disease that have been tested *in vivo* in the mouse model to date as further described in the text.

Chagas disease is a neglected disease that results from the kinetoplastid parasite *Trypanosoma cruzi* (*T. cruzi*)^1^ which infects 6–7 million people. The disease is endemic in Latin America but also has been identified in North America and Europe, primarily through immigration.^2−4^ The known clinical signs of disease are manifested in different phases. The acute phase lasts up to 8 weeks and can be asymptomatic or present flu-like symptoms, including fever, headache, and nausea. Past this time, the individual enters an asymptomatic chronic phase that is known to last for decades. About 25% of infected individuals will develop cardiomyopathy, and 5% of infected individuals will develop a digestive syndrome characterized by megacolon and/or megaesophagus. In both cases, the pathology is associated with inflammation of the muscle tissue and formation of fibrosis. Progression of clinical manifestations can ultimately lead to death, with organ transplantation being the only available treatment option. The spread of Chagas disease highlights the urgent need for effective therapeutics to treat infection, which are also safe and with novel mechanisms. The current pipeline for *T. cruzi* is sparse and lacks the necessary drug target diversity.^5−7^ The only available chemotherapies are benznidazole and nifurtimox. Benznidazole is approved for use in Chagas disease for children under the age of 12 in the U.S., but access is still limited.^8^ The treatment course is 60 days or more, with significant toxicity^9−11^ and controversial efficacy in the chronic disease stage.^12^ The results of the BENEFIT clinical trial^13,14^ showed benznidazole significantly reduced parasite burden, but these results were complicated because many patients had presented some evidence of cardiomyopathy at the time of enrolment. Overall, it did not prevent or reduce the progression of cardiopathy.^15^ Several molecules have progressed from animal models (Figure 1) to clinical trials without success^16−21^ including posaconazole^22^ and other CYP51 inhibitors.^23^ K11777 (identified by the Center for Discovery and Innovation in Parasitic Diseases at UCSD) targets cruzain, a validated target in preclinical studies,^24,25^ is also being developed to treat COVID-19, and has been considered safe in a Phase I clinical trial.^26^ Fexinidazole is in the clinical stage of development, sponsored by the Drugs for Neglected Diseases Initiative (DNDi),^27^ but since it is a nitro-heterocyclic compound like benznidazole and nifurtimox, the mechanism of action is likely to be the same.^28^ Several whole-cell, phenotypic high-throughput screens (HTS) have been reported for *T. cruzi*, including those at the Broad Institute,^29−31^ the Genomics Institute of the Novartis Research Foundation^32^ and GSK.^33^ These HTS are resulting in new hits^29−31,34−39^ from academia,^40^ industry, and the nonprofit sector, with the support of the National Institute of Allergy and Infectious diseases (NIAID), DNDi and others (e.g., proteasome inhibitors).^41,42^ Several recent studies also describe molecules with curative potential.^43,44^ Libraries of Food and Drug Administration (FDA) and European Union (EU)-approved drug or drugs approved by other jurisdictions can be virtually screened by such models to aid in drug repurposing.^45^

We have previously described how machine learning approaches alongside high throughput data from *T. cruzi* and *T. brucei* can be used to build machine learning models.^46^ These machine learning models were used to score and prioritize numerous drug libraries, and the top scoring 97 molecules were selected for purchase and testing. Five of these molecules (verapamil, pyronaridine, furazolidone, nitrofural and tetrandrine) demonstrated *in vitro* EC_50_ values less than 1 μM and were also tested for *in vivo* efficacy in a mouse model of Chagas disease that was dosed i.p.^47^ Pyronaridine showed 85.2% efficacy^47^ and was the most attractive to be pursued given its novelty against Chagas disease, and its approved status in Europe to treat uncomplicated malaria in combination with artesunate.^48,49^ The apparent mechanism of action of pyronaridine against *Plasmodium falciparum* appears to be through the targeting of hematin.^50^ Pyronaridine has also been proposed to inhibit DNA synthesis and replication, through intercalation and topoisomerase-2 inhibition at very high concentrations.^51,52^ Pyronaridine has been shown to have a lower toxicity as compared to compounds such as chloroquine.^53^ Knowing pyronaridine’s long half-life from previous malaria work,^53^ the compound was proposed as a potentially promising candidate for the further treatment of Chagas disease.^47^ We have now further explored this compound both as an oral (p.o.) monotherapy for acute and chronic therapy and in combination with benznidazole as a chronic combination treatment.

## Results and Discussion

### *In Vitro* Data on Different Parasite Strains

To investigate the susceptibility of various strains of *T. cruzi*, we performed an *in vitro* dose response efficacy test with pyronaridine with host cell toxicity being evaluated. We tested a reference strain (*T. cruzi* CA-I/72) alongside other *T. cruzi* strains. The reference strain *T. cruzi* CA-I/72 had a calculated IC_50_ of 0.159 μM (Figure S1A). We also tested for *in vitro* synergy between pyronaridine and benznidazole, but none was detected using the bliss algorithm as implemented in SynergyFinder 3.0 (Figure S2F). The combination of these molecules is additive or slightly antagonistic. Surprisingly, the calculated IC_50_ for pyronaridine against *T. cruzi* CL-luc was >10 μM (no antiparasitic activity observed up to the maximum tested concentration). We also tested a third strain (*T. cruzi* Colombiana) and obtained an IC_50_ of 0.21 μM (Table 1), although full antiparasitic activity was not seen in this strain (∼63%) (Figure S1B). The *in vitro* activity of pyronaridine against the *T. cruzi* Brazil-luc (Br-luc) strain was obtained by us previously^47^ and was found to have an IC_50_ 0.195 μM. Finally, we determined that the *in vitro* activity against the Sylvio X10/4 strain was shown to be similar to that found in CA-I/72, with an IC_50_ = 0.131 μM (Figure S1D) and similarly showed no *in vitro* synergy between pyronaridine and benznidazole in a checkboard assay (Figure S3F).

**Table 1:** Pyronaridine Antiparasitic Activity (IC_50_) in Different *T. cruzi* Strains Tested against Intracellular Parasites Infecting C2C12 Host Cells Assessed by Phenotypic Readout^a^

*Trypanosoma cruzi* strain	DTU	*In vitro* IC_50_ (μM)
CA-I/72 (reference strain)	TcI	0.16 ± 0.06
CL-luc	TcVI	>10
Columbiana	TcI	∼0.21
Brazil-luc	TcI	0.195^b^
Sylvio X10/4	TcI	0.13 ± 0.54

a See *in vitro* infection in the “Methods” section (*n* ≥ 2, ±SD).

b As described previously.^47^

### Pyronaridine Monotherapy and Combination Therapy in an Acute Mouse Model of Chagas Disease

**Figure 2:**
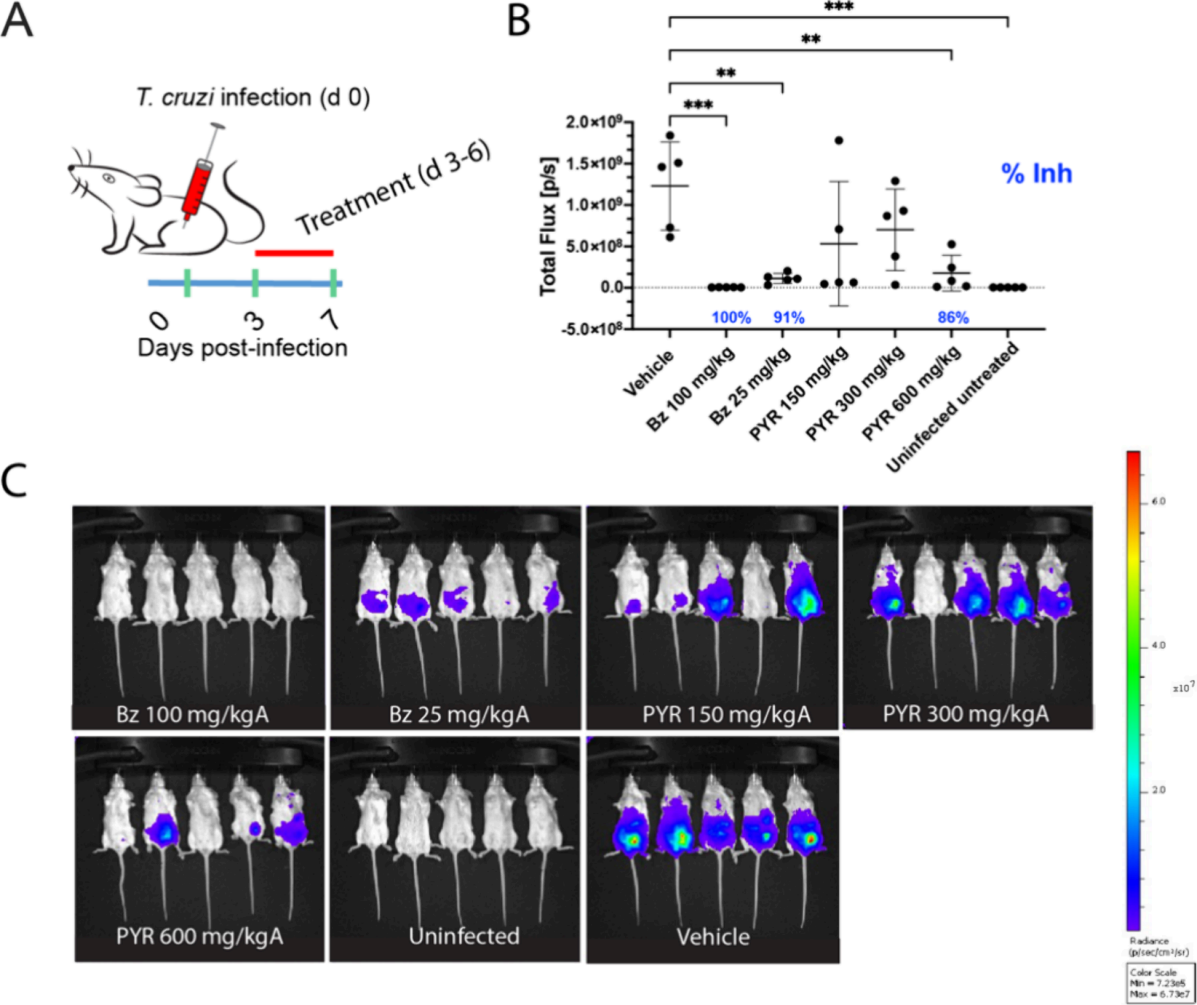
Acute mouse model of Chagas disease (*T. cruzi* Br-Luc). (A) Dosing schedule in mice. The green bars are the days the mice are read in the IVIS. Red bar represents the treatment period (bid). (B) *In vivo* antiparasitic activity (day 7 postinfection) following oral dosing of pyronaridine (PYR) or benznidazole (Bz). (C) Luminescent signal in mice following the treatment with different drugs or vehicle. Statistical significance was determined using an ordinary one-way ANOVA with Dunnett’s multiple comparisons test as calculated in Graphpad Prism 10.0. Only comparisons that are statistically significant (* *p* ≤ 0.05, ** *p* ≤ 0.01, *** *p* ≤ 0.001, **** *p* ≤ 0.0001) are shown. Percent inhibition is calculated from the mean of each group as compared to the vehicle and only significant inhibition is shown.

We first assessed the efficacy of orally administered pyronaridine as a monotherapy against *T. cruzi* CL-luc in the acute mouse model of Chagas disease and observed a lower parasite burden reduction after treatment than what was previously shown against Br-luc when dosed i.p.^47^ (Table S1). Combined with the results from the *in vitro* assay (Table 1), we concluded that the CL-luc parasite was less sensitive to pyronaridine than other strains and therefore repeated the experiment using the Br-luc parasite strain. We dosed groups with either 50 mg/kg benznidazole (control), 100 or 150 mg/kg pyronaridine administered via oral gavage, and an i.p.-administered 50 mg/kg pyronaridine for comparison to an earlier study^47^ (Figure S4). The results showed a 93% burden reduction when dosed i.p., but while there was a trend in a parasite burden reduction at 100 and 150 mg/kg oral doses, this was not statistically significant compared to the untreated control group. Further investigation indicated that oral pyronaridine had a similar oral bioavailability as compared to i.p. in mice (Figure S5), and hence the reason for this difference is unknown. Oral administration offers more clinical advantages, so we altered the dosing to treat *T. cruzi* Br-luc infected mice with 150, 300, and 600 mg/kg pyronaridine p.o.. The results then showed a statistically significant 86% reduction of parasites at day 7 when pyronaridine was dosed p.o. 600 mg/kg (Figure 2).

**Figure 3:**
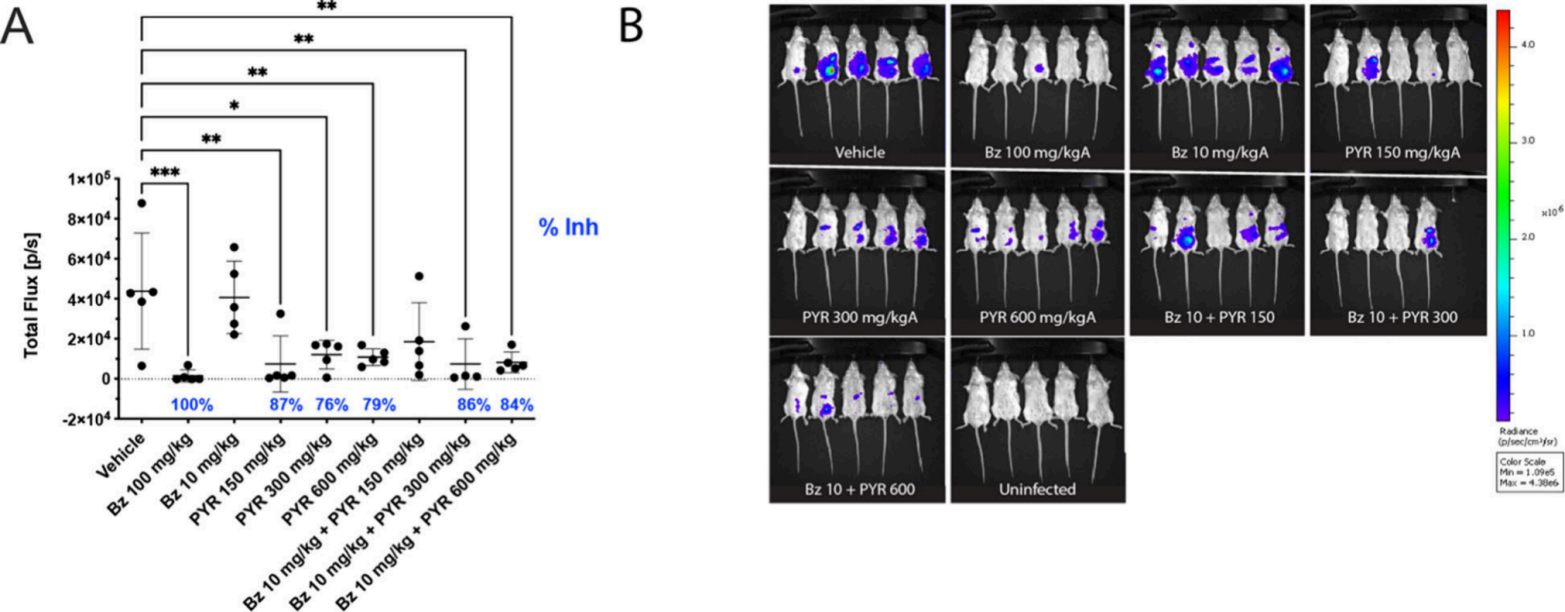
Acute mouse model of Chagas disease (*T. cruzi* Br-Luc) after combination therapy with benznidazole and pyronaridine. (A) *In vivo* antiparasitic activity (day 7 postinfection) following oral dosing of pyronaridine (PYR) and or benznidazole (Bz). (B) Luminescent signal in mice following the treatment with different drugs or vehicle. Statistical significance was determined using an ordinary one-way ANOVA with Dunnett’s multiple comparisons test against the vehicle as calculated in Graphpad Prism 10.0. Only comparisons that are statistically significant (* *p* ≤ 0.05, ** *p* ≤ 0.01, *** *p* ≤ 0.001) are shown. Percent inhibition is calculated from the mean of each group as compared to the vehicle and only significant inhibition is shown.

The efficacy of combination therapy with benznidazole and pyronaridine was also investigated in the acute model with Br-luc parasites in order to determine the optimal dosage combinations for eventual use in the chronic model. Conceptually, this would allow the use of much lower doses of benznidazole, theoretically thereby reducing the known toxicity associated with this drug. First, we tested to determine suboptimal doses of benznidazole in the acute model of Chagas disease (Figure S6). 10 mg/kg benznidazole dosed p.o. was chosen as it was found to have a modest 39% parasite inhibition. Following this, we tested 10 mg/kg benznidazole combined with either 150, 300, or 600 mg/kg pyronaridine p.o. Interestingly, the 10 mg/kg benznidazole control only resulted in an 11% parasite inhibition in this combination study, but when combined with pyronaridine at 300 mg/kg it resulted in 86% inhibition. The combination of the two drugs was therefore effective in reducing the benznidazole concentration and still clearing parasites in a statistically significant manner in the acute model (Figure 3). It is important to note combining both drugs was not to try to improve efficacy of treatment. We selected the 10 mg/kg benznidazole and 300 mg/kg pyronaridine p.o. as the combination to test in the chronic model of Chagas, to investigate if this treatment could prevent cardiac symptoms.

### Pyronaridine Monotherapy and Combination Therapy in a Chronic Mouse Model of Chagas Disease

**Figure 4:**
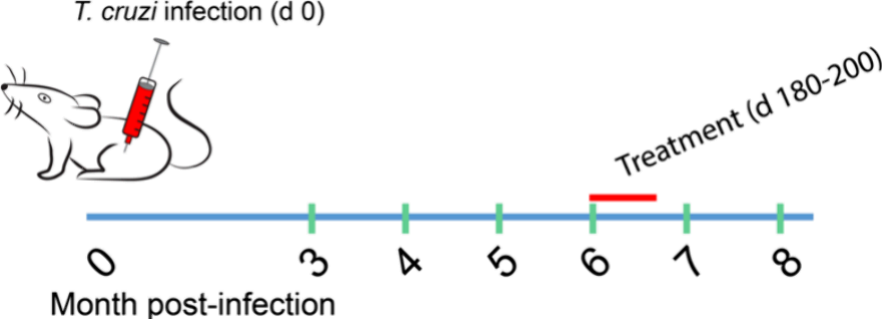
Chronic mouse model of Chagas disease. The green bars are the days EKG were performed for heart condition assessment. Red bar represents treatment.

Our next step was to perform the combination study in the chronic model to assess if it would have efficacy *in vivo* (Figure 4). For the chronic mouse model of Chagas disease, we utilized the model that was developed in our laboratory previously^54^ using the validated *T. cruzi* Sylvio X10/4 parasite strain,^22,55^ which we showed is susceptible to pyronaridine *in vitro* (Table 1). Briefly, from a cohort of 40 C57/B16 mice that were 7 weeks old, we infected 35 animals with 1 × 10^6^*T. cruzi* Sylvio X10/4 trypomastigotes by i.p. injection, with 5 noninfected controls. At three months postinfection, we performed an EKG analysis in all individuals to assess cardiac status. Three mice from the infected group died of natural causes (possibly unrelated to the infection since the challenge would not be expected to be lethal). We divided the remaining animals into the following 7 groups and started treatment 6 months postinfection as follows: uninfected vehicle (10% Solutol, 5 mice), infected vehicle (10% Solutol, 7 mice), benznidazole 100 mg/kg (monotherapy, 5 mice), benznidazole 10 mg/kg (suboptimal dose, 5 mice), pyronaridine 300 mg/kg (monotherapy, 5 mice), pyronaridine 300 mg/kg + benznidazole 10 mg/kg (combination therapy, 5 mice), and infected/untreated (5 mice).

**Figure 5:**
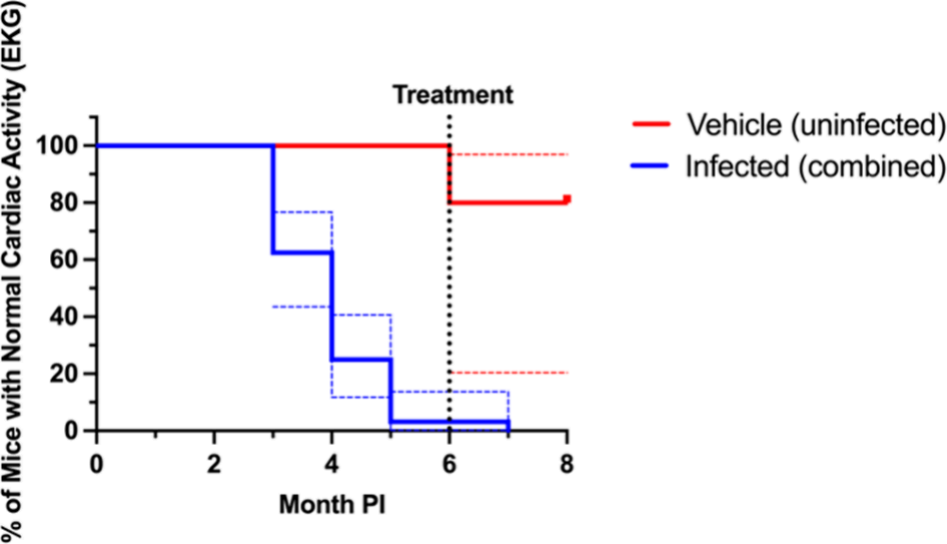
Kaplan–Meier graph of cardiac abnormalities from the chronic mouse model of *T. cruzi* Sylvio X10/4 infection model data. An event is defined as an abnormal EKG. For experimental groups, treatment began at 6 month PI so for clarity all infected groups were combined into a single “infected group”. Blue and red dotted lines represent the 95% confidence intervals to show the variability in infected groups. PI = postinfection.

The mice were then orally dosed daily for 20 days with specific doses per group as described. We monitored the cardiac function by electrocardiogram (EKG) at months 3, 4, 5, 6, 7, and 8. The results are illustrated in Table S2, Figure S7, and Figure 5 (Kaplan–Meier analysis); all infected groups show abnormal EKG data (Figure S8) at these time points with a reduction in the Q-T Interval from 6 months postinfection to 7 and 8 months postinfection. The mice had a final cardiac assessment 8 months postinfection, just before being euthanized. During the last EKG measurement, 3 mice from Group 6 (combination benznidazole and pyronaridine) died after receiving anesthesia for the procedure. Therefore, for the final measurements in the combination therapy group, we considered the results from the two remaining animals.

**Figure 6:**
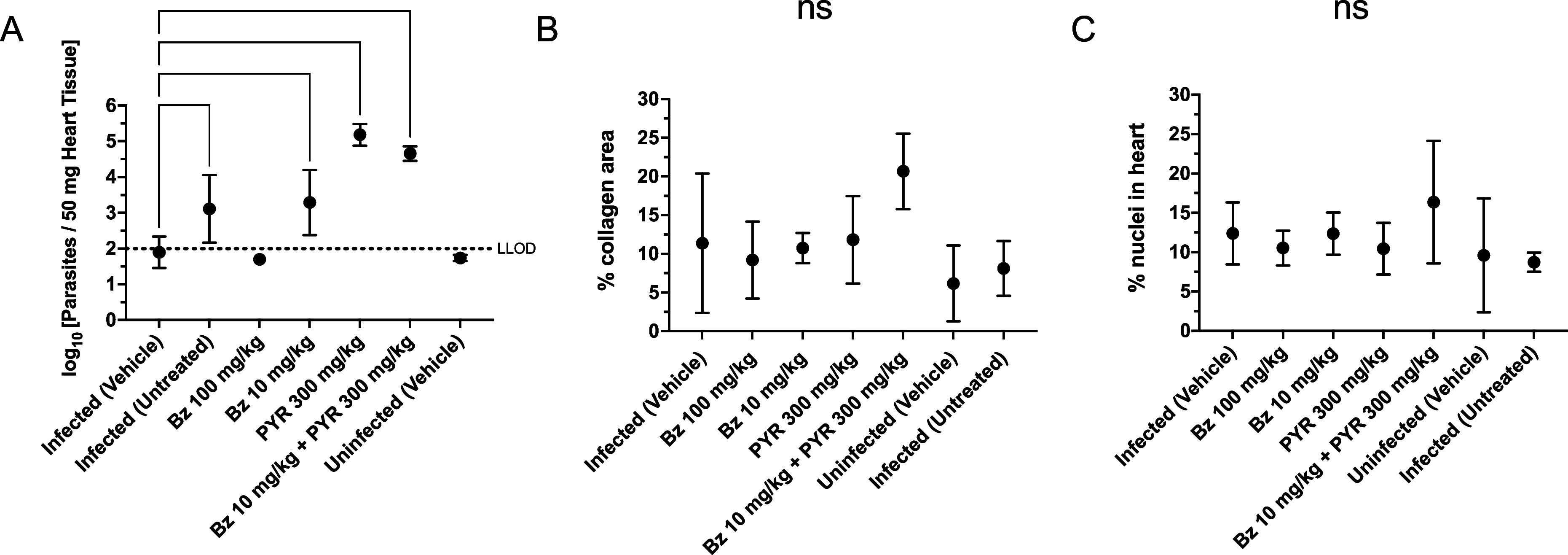
Results for from the chronic study against *T. cruzi* Sylvio X10/4. Parasite burden: (A) RT-PCR results from the collected heart samples. Histological: (B) heart fibrosis measured as percent collagen area and (C) heart inflammation based on histology analysis. Statistical significance of the data (log transformed for RT-PCR) was determined using an ordinary one-way ANOVA with Dunnett’s multiple comparisons test against the infected vehicle as calculated in Graphpad Prism 10.0.2. Only comparisons that are statistically significant (* *p* ≤ 0.05, ** *p* ≤ 0.01, **** *p* ≤ 0.0001) are shown. Additional comparisons are shown in Figure S8. Bars represent SD from the mean (line). No statistically significant difference (ns) was found for heart fibrosis (B) or heart inflammation (C) from the infected control (vehicle-treated). For RT-PCR, all values below the lower limit of detection (LLOD) are set to 0.5 × LLOD.

In addition, we collected hearts to perform real-time polymerase chain reactions (RT-PCRs) to quantify traces of parasites and to perform histology to assess if any of the treatments could prevent cardiac pathology. The results from the RT-PCR targeting *T. cruzi* parasites (Figure 6A) showed that the groups with higher numbers of parasites per tissue were the mice treated with pyronaridine (300 mg/kg) and the combination benznidazole (10 mg/kg) and pyronaridine (300 mg/kg), followed by benznidazole (10 mg/kg) and infected/untreated control group. A group by group statistical comparison was also done with multiple groups showing statistically significant differences (Figure S9). It is important to mention that the limit of detection for the assay is ∼100 copies; therefore, anything below that can be considered negative (absence of *T. cruzi* in the sample). The results suggested that neither pyronaridine as a monotherapy at 300 mg/kg nor the combination of pyronaridine with benznidazole had any efficacy at killing the parasites in this chronic model. However, it should be mentioned that only 2 mice remained in the combination group at the end of the study. Only benznidazole at 100 mg/kg demonstrates a level of parasites in the tissue below the level of detection, as was also reported earlier in a similar chronic model using the Br-luc strain.^22^ The next step was to look at the tissue status. We assessed fibrosis using the collagen area as a readout (Figure 6B) and observed no significant change in collagen areas comparing groups to untreated or uninfected controls using *t* test or one way ANOVA. Comparing the levels of cardiac inflammation in the different groups (Figure 6C), again we found no significant difference between the treated groups and infected and uninfected vehicle controls groups using *t* test or one way ANOVA.

The benefits of antitrypanosome therapy are well established in the acute phase of Chagas disease. The two nitroheterocyclic drugs described in the 1960s and 1970s, nifurtimox and benznidazole, have shown clear antiparasitic effects, with elimination of both the circulating and tissue forms of the parasite when administered in the acute phase of Chagas disease.^56,57^ However, in the chronic form of the disease, in which the role of the parasite is not as well understood, there is controversy as to whether its eradication is beneficial.^58^ The data supporting specific antiparasite therapy includes evidence of vestiges of parasites that can be identified by PCR in the inflamed cardiac tissue during the chronic phase of human infections, as well as evidence of the antitrypanosomal therapy reducing the inflammatory burden in the cardiac tissue in experimental animal models.^59−62^ Currently, benznidazole treatment is limited only to those who tolerate the drug, with no rescue therapy available for those who fail this treatment. Benznidazole treatment in the early phase of the chronic form of the disease has shown encouraging results, with negative seroconversion achieved in 58–62% of the cases after 3–4 years of follow-up in infected children.^56,63^ In addition, a nonrandomized, nonblinded study showed a noticeably slower progression to severe cardiomyopathy in adults that were receiving benznidazole.^64^ Finally, a published systematic review of five clinical trials, which studied 756 patients, suggested that benznidazole can reduce parasite-related outcomes in Chagas cardiomyopathy and can also result in negative xenodiagnosis and higher rates of seroreversion.^65^ The tolerance of benznidazole is inversely related to the patient’s age, with adverse effects being quite uncommon in children but occur in 30–50% of adult patients and include severe dermatitis, polyneuritis, and depression of bone marrow function.^11,65^ The absence of any robust evidence to support the universal treatment of all *T. cruzi* seropositive patients with benznidazole highlighted the need for developing safer, more efficacious drugs, particularly in those patients with indeterminate and chronic cardiomyopathy forms of the disease.^66,67^

A comprehensive review of the antimalarial properties and product characteristics of pyronaridine has been published^53^ describing its use as a monotherapy in clinical trials for malaria,^68^ while pyronaridine and artesunate is combined in a tablet formulation and is approved for *P. falciparum* and *Plasmodium vivax* blood stage malaria.^69,70^ Pediatric formulations have also been tested in combinations for malaria and it appears it is well tolerated with no serious toxicities.^71^ The pharmacokinetics of pyronaridine has been assessed in rat, rabbit, dog, rhesus monkey, and humans. Rat and dog showed a terminal half-life of 2–4 days after IV dosing, while rabbit and rhesus monkey showed an apparent half-life of 2–3 days.^53^ In the rat, the half-life of elimination is 137–231 h, and in humans the half-life is 241 h. Pyronaridine pharmacokinetics in mice and guinea pig were recently reported by us suggesting a longer half-life in mouse.^72,73^ We have previously identified that pyronaridine can statistically significantly decrease lung inflammation and modify levels of several cytokines in a mouse model infected with SARS-CoV-2.^74^ These effects of pyronaridine on cytokines and inflammation may be relevant to this study and other pathogens.

We previously identified the *in vitro* and *in vivo* activity of pyronaridine against *T. cruzi* (CA-I/72 and Brazil-luc, respectively)^47^ and described an EC_50_ of 4.6 μM against the Tulahuen strain of the parasite.^75^ In the current study, we noticed strain differences (as highlighted by CL-*luc*) that merit future investigation of strain-dependent resistance or susceptibility to pyronaridine. This level of susceptibility to compounds is not common for this parasite, which raises the following question: is the resistance caused by an intrinsic property of the CL-*luc* parasite, or is it related to the expression of the red-shifted luciferase protein (or associated with the expression system used)? It may also be important in future studies to understand whether this effect is seen across other strains. While the mechanism of action of pyronaridine against *T. cruzi* is unclear, it may also have immunomodulatory effects similar to studies in peripheral blood mononuclear cells from Chagas disease patients with K777,^75^ based on the cytokine responses observed in mice previously treated with pyronaridine for other diseases.^72,74^

In summary, the current study indicates that oral pyronaridine, which has been used safely for decades in the treatment of malaria, has activity against acute Chagas disease in mice when dosed orally. In contrast, in the chronic mouse model with the Sylvio X10/4 strain it is not effective as it results in a significant increase in parasite burden in pyronaridine-treated animals. This may be due to the inability of pyronaridine to achieve a concentration above the *in vitro* IC_50_ in the heart, which would require follow-up studies to confirm this. This is not unprecedented, as differences between the acute (Sylvio X10/7) and chronic (Brazil) model performance were also observed previously for posaconazole treatment.^22^ While this study did not demonstrate any pyronaridine activity in the chronic phase of Chagas disease and was not able to reverse cardiomyopathy, changes in the strain, administration frequency, or duration may yield different results. We consider that these findings are nonetheless noteworthy and should be shared with the scientific community.

## Methods

### Chemicals and Reagents

Pyronaridine tetraphosphate (P0049–250MG, Lot # MKCH3442, animal studies acute study TC190816 and chronic study TC200430, MKCB5984), benznidazole (#41965–1G Lot MKCD5602) and Bouins solution (Sigma HT10132–1L) were purchased from Sigma-Aldrich. Quick-DNA Miniprep Plus kit (catalog no. D4068, lot 207598), ZR Bashing Bead Lysis tube, 2.0 mm beads (catalog no. 56003–50, lot 430251), and DNA/RNA shield (catalog no. R1100–50, lot 196122) were purchased from Zymo Research. Fast SYBR Green Master Mix was purchased from Applied Biosystems (catalog # 4385610).

### Cells and Parasites

Mouse C2C12 myoblasts (ATCC CRL-1772) were cultivated in RPMI 1640 medium supplemented with 5% FBS and kept at 37 °C with 5% CO_2_. All strains of *T. cruzi* were maintained in a C2C12 myoblast culture. After 5–7 days of passage of cells and parasites, trypomastigotes released in the supernatant were collected for C2C12 reinfection and propagation of culture up to 20 passages, when new stocks were used to restart the culture. *T. cruzi* CA-I/72 and Sylvio-X10/4 were kindly provided by Dr. James Dvorak.^76,77^*T. cruzi* Br-luc was kindly provided by Dr. Ana Rodriguez,^39^ and *T. cruzi* CL-luc was kindly provided by Dr. John Kelly.^78^

### *In Vitro* Infection

*T. cruzi* trypomastigotes from different strains (Brazil-luc, Cl-luc, Sylvio-X10/4, CA-I/72, and Colombiana) were obtained from the supernatant of C2C12 cells infected 4–7 days previously. CA-I/72,^79^ Sylvio-X10/4,^80^ Colombiana,^80^ represent strains from DTUs TcI while CL-luc and Brazil-luc are TcVI^81^ and TcI,^39,82^ respectively, so some diversity is represented. For the assay, 500 C2C12 cells were mixed with 7,500 *T. cruzi* parasites and seeded into 384-well plates at a 50 μL final volume. Test compounds (benznidazole Sigma cat. no. 419656 and/or pyronaridine Sigma cat. no. 04277) were prespotted into the 384-well black bottom plates in different concentrations using an Acoustic Transfer System (ATS, EDC Biosciences) prior to the addition of cells and parasites. The plates were incubated at 37 °C, 5% CO_2_ for 48 h. The plate content was then fixed with 4% formaldehyde, and 0.5 μg/mL DAPI (diamidino-2-phenylindole) was added for staining of the nucleic acids. Plates were kept protected from light for at least 1 h and then imaged in an ImageXpress Micro XLS automated high content imager (Molecular Devices, San Jose, CA, USA). Imaging and data analysis were performed using GraphPad Prism 9 software with biological triplicates.

To examine the combined antiparasitic activity of pyronaridine and benznidazole (Sigma-Aldrich) against *T. cruzi* CA-I/72 and Sylvio-X10/4 trypomastigote parasites, both compounds were spotted into 384-well plates (Greiner) using an Echo 650 Acoustic Liquid Handler (Beckman Coulter). Pyronaridine and benznidazole were dissolved in DMSO and plated in dose–response curves using a 10-point 2-fold serial dilution, starting at 2.5 μM for pyronaridine and 40 μM for benznidazole, following a checkerboard combination assay as described elsewhere (*n* = 4).^83,84^ Controls (*n* = 12) containing only pyronaridine or only benznidazole were included, as well as only DMSO. C2C12 murine cardiomyoblast cells (ATCC CRL-1772) were seeded at 700 cells/well, and *T. cruzi* tissue-culture trypomastigotes were added at a ratio of 10 parasites/cell for the CA-I/72 strain. Both cells and parasites were plated in DMEM High-Glucose medium (Gibco, cat. no. 11995065) containing 5% fetal bovine serum (Sigma-Aldrich, cat. no. F2442) and 1% penicillin-streptomycin (Gibco, cat. no. 15140122). Cells and parasites were incubated in the presence of the compounds for 48 h at 37 °C and 5% CO_2_. Plates were then fixed with 4% formaldehyde solution for at least 1 h, washed with 1× PBS and stained with 5 μg/mL DAPI. Plates were read using an ImageXpress microscope and analyzed by MetaXpress software (Molecular Devices) using a custom module optimized for this assay. The same checkerboard combination assay was performed against *T. cruzi* Sylvio-X10/4 trypomastigote parasites, but instead using 20 parasites per cell and incubating the plates for 72h. Data was further analyzed using SynergyFinder 3.0.^85^

### Pharmacokinetics

Pyronaridine tetraphosphate from Sigma-Aldrich (P0049–250MG, lot no. MKCB5984) was administered to six-week-old female BALB/c/J mice. Each mouse was dosed with a single bolus dose of pyronaridine tetraphosphate in 20% Solutol at 50 mg/kg (1 mg/mouse in 100 μL). Administration was either via oral gavage or i.p. injection (*n* = 2). Plasma was evaluated from blood samples collected before dosing (prebleed; *t* = 0) and 0.5, 2, 4, 8, and 20 h postdose.

### Chagas Disease Acute Infection Model

Trypomastigote parasites collected from maintenance culture were used for *in vivo* infection after centrifugation for 15 min (min) at 3300 rpm, resuspended in Dulbecco’s modified Eagle’s medium (DMEM). BALB/c mice (6-weeks old, female) were infected intraperitoneally with 1 × 10^6^ of the trypomastigote form of *T. cruzi* Brazil-luc^39^ or 1 × 10^3^*T. cruzi* Cl-luc^86^ per mouse. The treatment started 3 days postinfection and was carried out for 4 consecutive days. At 7 days postinfection, the mice were anesthetized with isoflurane and imaged in the *In Vivo* Imaging System (IVIS, PerkinElmer) after administration of 100ul of 5 mg/mL luciferase solution (d-Luciferin potassium salt from GoldBio (cat. no. LUCK-1G)). It was injected intraperitoneally and imaged in IVIS between 10 and 20 min after injection (prior studies showed the luciferase signal is detectable after even 3 min postinjection, peaks at ∼8 min, and stays constant for more than 30 min before decaying slowly). The luminescence signal read from the instrument correlated to the parasite infection burden. The relative luminescence per animal was quantified and normalized based on infected/untreated controls and noninfected controls.

### Chagas Disease Chronic Infection Model

37-week-old female C57BL/6J mice (The Jackson Laboratory) infected with 1 × 10^6^*T. cruzi* Sylvio X10/4 trypomastigotes in 100 μL of DMEM (without FBS or antibiotics) by i.p. injection and were divided into 6 groups of 5 mice per group, and an additional group of 5 age and sex matched uninfected mice was included as a control and kept under the same conditions. Electrocardiography was performed at the Seaweed Canyon Cardiovascular Physiology Laboratory, Institute for Molecular Medicine, UCSD monthly starting at 3 months postinfection followed by analysis (PowerLab ADInstruments Chart Module Series; product MLS360 EKG analysis module) to monitor the development of heart disease. Beginning at 6 months postinfection, mice were dosed orally once a day for 3 weeks with either vehicle (20% Kolliphor HS 15, Sigma-Aldrich), benznidazole (100 mg/kg), benznidazole (10 mg/kg), pyronaridine (300 mg/kg), or a combination of benznidazole (10 mg/kg) and pyronaridine (300 mg/kg). A group of infected untreated mice was also included in the study. In addition, a group of uninfected mice were dosed with vehicle following the same dosing schedule. The infected groups were age and sex matched with uninfected controls and kept under the same conditions. The general health of the mice was evaluated weekly. After the final EKG was performed 8 months postinfection, animals were euthanized by exposure to CO_2_ in an approved chamber immediately followed by cervical dislocation and tissue collected for further analysis.

### Surface EKG

Adult mice were anesthetized with isoflurane (5% induction, 1–1.5% maintenance in 100% oxygen) and placed on a warming pad (35 °C– 37 °C). Needle electrodes made of 27-gauge needles were inserted subcutaneously into each of the four limbs and the chest area. Simultaneous standard EKG leads I and II and chest leads were recorded at a frequency response of 0.05–500 Hz. The signal was digitized and recorded at 2,000 Hz on LabChart (ADinstruments). The PowerLab ADInstruments Chart Module Series; product MLS360 EKG analysis module was used for data analysis. Sample EKGs are shown for 7 and 8 months postinfection for the chronic study in Figure S8.

### Histology

Upon euthanasia, the hearts were collected from the mice and cut in half in the sagittal orientation, placed in cryomolds, embedded in Tissue-Tek (O.C.T., Sakura Finetek), and frozen on dry ice, and the frozen tissue blocks were stored at −80 °C. A cryostat (Leica CM 1850) was used to cut tissue sections (10 μm) from frozen tissue blocks, which were mounted on Fisher brand Superfrost plus slides (Fisher Scientific cat. no. 12–550–15) and stored frozen at −20 C until stained.

Fibrosis: Collagen staining was done using the Sigma-Aldrich Trichrome Stain (Masson’s) Kit (Sigma-Aldrich, catalog no. HT15) with Weigart’s Iron Hematoxylin (Sigma, Cat. # HT1079). The slides were scanned using Nanozoomer Slide Scanner (Hamamatsu Photonics, NJ, USA) and images were obtained through NDP viewer software (Hamamatsu Photonics, NJ, USA).

Inflammation: Hematoxylin and eosin staining was used to measure the number of nuclei in the tissue.

### Histopathology Analysis

As described previously^22,87^ inflammation was quantified using 5 random images of mouse heart (magnification 10×) from each animal representing most of the section.

### qPCR

Tissue (50 mg) was homogenized with DNA/RNA shield (Zymo Research, cat. no. R1100–50) in ZR Bashing Bead Lysis tubes (Zymo Research, cat. no. 56003–50) according to the manufacturer’s instructions. DNA was extracted from 50 mg of homogenized tissue slices using the Quick-DNA Miniprep Plus kit from Zymo Research (Cat. # D4068). DNA was quantified by nanodrop and 180 ng were used for qPCR using Fast SYBR Green Master Mix (Applied Biosystems, Cat. # 4385610) on a Stratagene Mx3005P RT-PCR thermocycler. qPCR primers were ASTCGGCTGATCGTTTTCGA and AATTCCTCCAAGCAGCGGATA to amplify the parasite satellite DNA region and TCCCTCTCATCAGTTCTATGGCCCA and CAGCAAGCATCTATGCACTTAGACCCC to amplify host TNFα2 using the following thermal profile: initial denaturation at 95 °C for 10 min, then 40 cycles of denaturation at 95 °C for 30 s, annealing at 55 °C for 60 s, and extension at 72 °C for 60 s. Followed by 1 cycle of 95 °C for 60 s, 55 °C for 30 s, and 95 °C for 30 s. Melting curve analysis was used to confirm the correct PCR product formation. A standard curve was generated from samples extracted from mouse heart tissue spiked with 2 × 10^7^*T. cruzi* epimastigotes and was used to determine parasite burden in each tissue.

### Statistical Analysis

Statistical analysis was performed using Graphpad Prism (GraphPad Software, San Diego, California USA).

### Ethics Statement

Use of mice was in accordance with a protocol approved by UC San Diego’s Institutional Animal Care and Use Committee. The committee derives its authority for its activities from the United States Public Health Service (PHS) Policy on Humane Care and Use of Laboratory Animals and the Animal Welfare Act and Regulations (AWAR). All animal studies were performed under approved protocol S14187 from the Institutional Animal Care and Use Committee, University of California, San Diego (AAALAC Accreditation Number 000503) and in compliance with the Animal Welfare Act and adheres to the principles stated in the Guide for the Care and Use of Laboratory Animals, National Research Council, 2011.

## Supporting Information Available

Details on *in vitro* and *in vivo* antiparasitic activity, pharmacokinetics data in mice, suboptimal dosing, mouse weights in the chronic infection study, comparisons of *in vivo* efficacy studies, cardiac function assessment from the chronic infection study (PDF)

## Abbreviations

ADME: absorption, disposition, metabolism and excretion
NIAID: National Institute of Allergy and Infectious diseases
ANOVA: analysis of variance
b.i.d.: twice a day
DNDi: Drugs for Neglected Diseases initiative
DMEM: Dulbecco’s modified Eagle’s medium
FDA: Food and Drug Administration
EKG: Electrocardiogram
EU: European Union
RT-PCR: real-time polymerase chain reaction
i.p.: intraperitoneal
p.o.: oral
*Trypanosoma cruzi*: *T. cruzi*
