# Supplementary material for: Oral Pyronaridine Tetraphosphate Reduces Tissue Presence of Parasites in a Mouse Model of Chagas Disease
Source: ACS Omega. 2024 Aug 20;9(35):37288–98. doi: 10.1021/acsomega.4c05060 (PMC11375811; doi:10.1021/acsomega.4c05060)
Supplement: Supplementary file 1 — ao4c05060_si_001.pdf [file ao4c05060_si_001.pdf]

Supplemental data

Oral Pyronaridine Tetraphosphate Reduces Tissue Presence of Parasites in a Mouse Model of Chagas Disease

Jair Lage Siqueira-Neto[1*], Thomas R. Lane[2], Jean A. Bernatchez[1], Claudia Magalhaes Calvet Alvarez[1,3], Elany Barbosa da Silva[1], Miriam A. Giardini[1] and Sean Ekins[2*]

[1]Center for Discovery and Innovation in Parasitic Diseases, Skaggs School of Pharmacy and Pharmaceutical Sciences, University of California, San Diego, La Jolla, CA, USA [2]Collaborations Pharmaceuticals, Inc., 840 Main Campus Drive, Lab 3510, Raleigh, NC 27606, USA. [3]Laboratório de Ultraestrutura Celular, Instituto Oswaldo Cruz, FIOCRUZ, Rio de Janeiro, RJ, Brazil, 21040-300

[*]To whom correspondence should be addressed: Jair Lage de Siqueira-Neto E-mail address: jlagedesiqueiraneto@health.ucsd.edu Sean Ekins, E-mail address: sean@collaborationspharma.com

Number of pages (18), figures (7) , and tables (2).

Short running title: Pyronaridine *in vivo* efficacy against Chagas Disease

Figure S1. Pyronaridine antiparasitic activity (IC_{50}) was assessed by phenotypic analysis of infected C2C12 with different *T. cruzi* strains as described in the Methods section. (n≥2, ±SD, error bars represent SD*).* IC_{50}s are calculated using a 4-parameter dose response curve fit using Graphpad Prism 10.0.2. SDs of IC_{50}/CC_{50} are shown when calculatable.

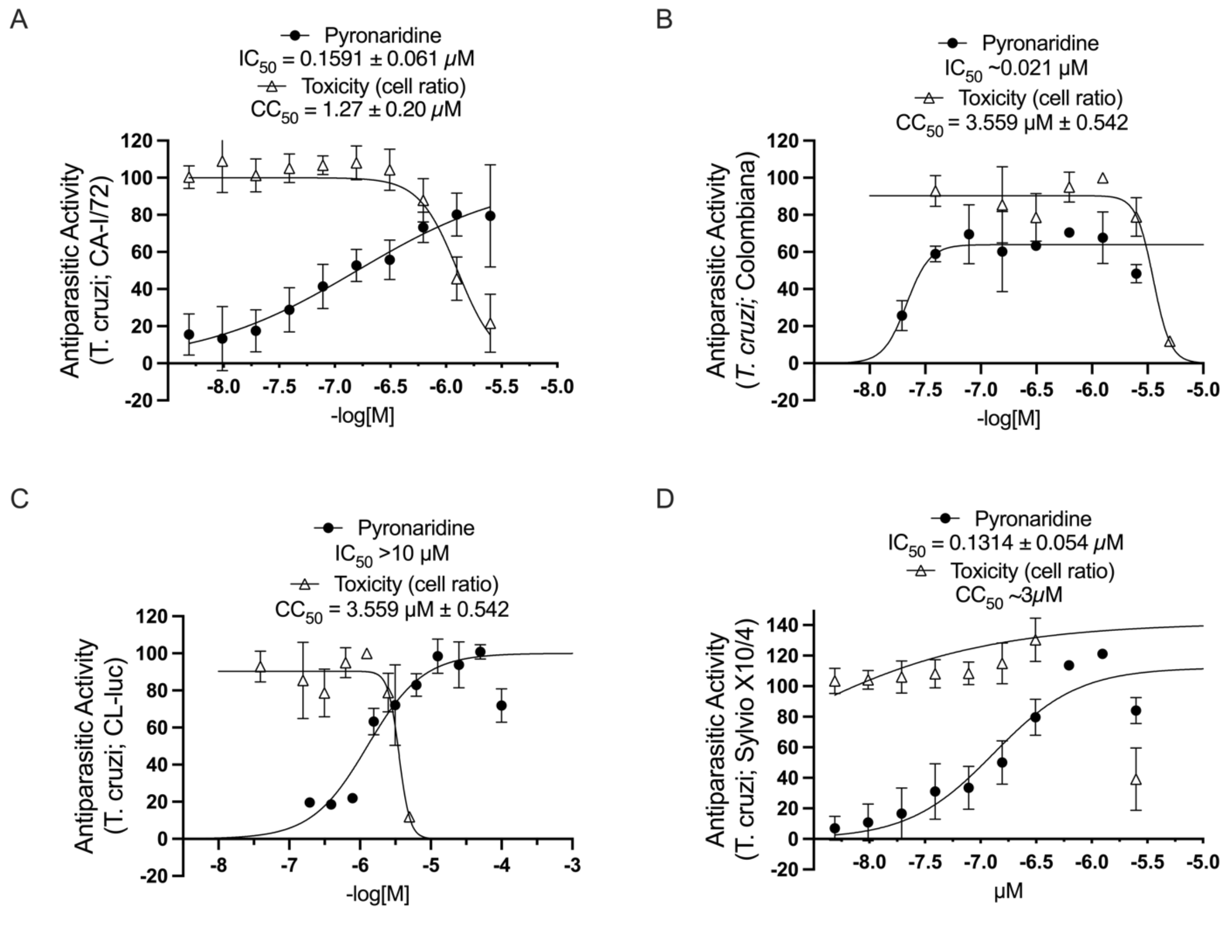

Figure S2. Antiparasitic synergy analysis (SynergyFinder 3.0) of a checkerboard assay with pyronaridine and benznidazole activity as assessed by a phenotypic analysis of infected C2C12 with *T. cruzi* strain CA-I/72. Dose-response plots of the inhibition (A, C) and cell toxicity (B, D) of the individual drugs from the checkboard assay as fitted by four-parameter logistic curve (n=12). IC_{50}s and CC_{50}s were calculated using Prism 10.2 (4-parameter fit, ± SD). The mean response, with SD shown below, of each condition for inhibition (E) and cell toxicity (F). Synergy determination was done using the bliss algorithm as implemented in SynergyFinder 3.0. The 2D synergy maps (G, H) highlight synergistic and antagonistic dose regions in red and green colors, respectively. Based on their criteria: x < -10 antagonistic; -10≥ x ≥10 additive; >10 synergistic, defining PYR and BZ as not synergistic.

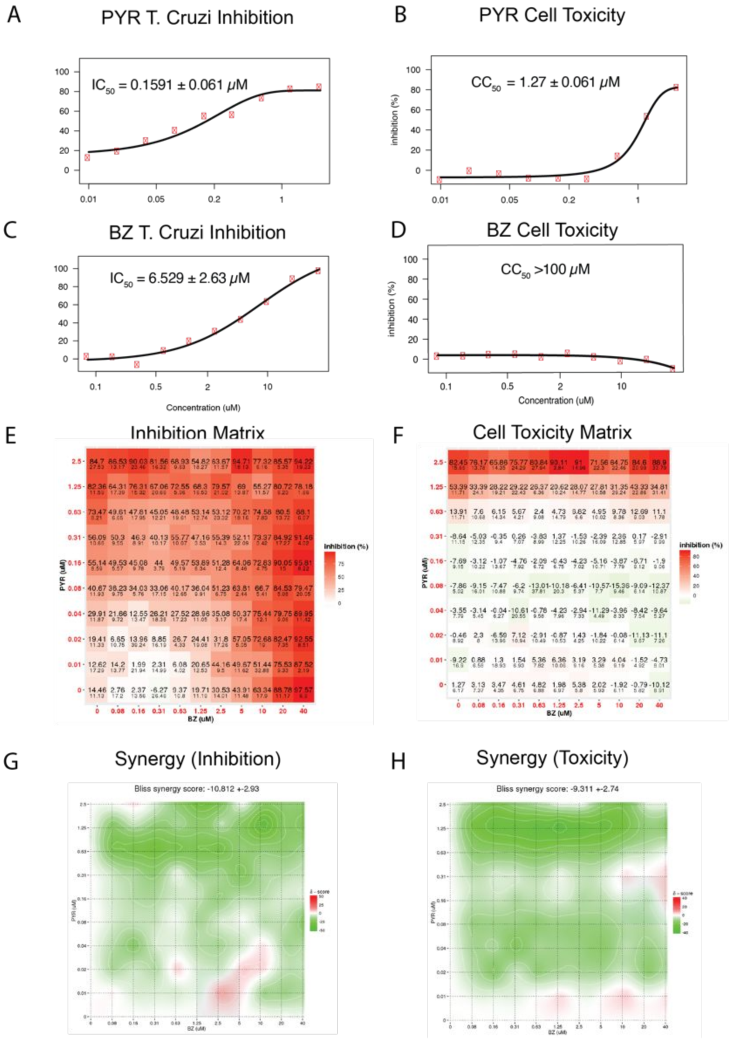

Figure S3. Antiparasitic synergy analysis (SynergyFinder 3.0) of a checkerboard assay with pyronaridine and benznidazole activity as assessed by a phenotypic analysis of infected C2C12 with *T. cruzi* strain Sylvio X10/4. Dose-response plots of the inhibition (A ,C) and cell toxicity (B, D) of the individual drugs from the checkboard assay as fitted by four-parameter logistic curve (n=12). IC_{50}s and CC_{50}s were calculated using Prism 10.2 (4-parameter fit, ± SD). The mean response, with SD shown below, of each condition for inhibition (E) and cell toxicity (F). Synergy determination was done using the bliss algorithm as implemented in SynergyFinder 3.0. The 2D synergy maps (G, H) highlight synergistic and antagonistic dose regions in red and green colors, respectively. Based on their criteria: x < -10 antagonistic; -10≥ x ≥10 additive; >10 synergistic, defining PYR and BZ as not synergistic.

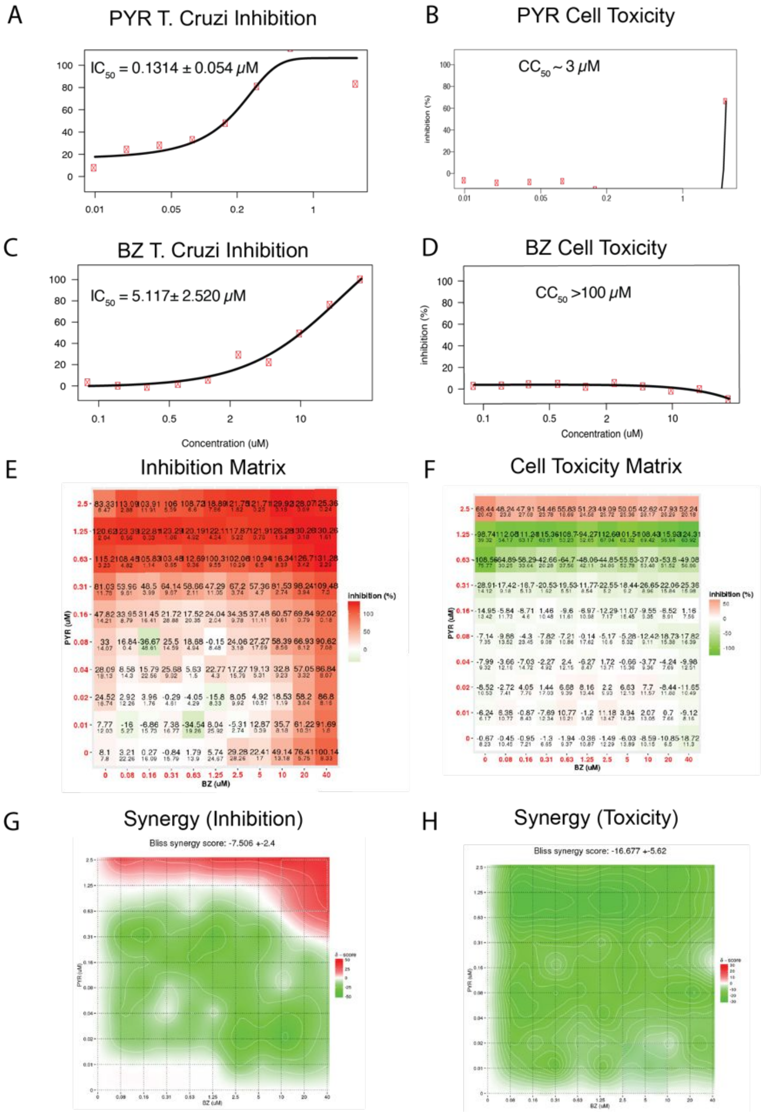

Figure S4. Antiparasitic activity of oral pyronaridine (PYR) or benznidazole (Bz) against *T. cruzi* br-luc 7 days post infection following treatment a three-day (days 3-6) treatment of PYR (oral or i.p.) or Bz (oral). Comparison of total flux is represented graphically (A) based on the luminescent signal in mice following treatment with different drugs or vehicle (B, C). Total flux analysis is sex independent, but samples are separately shown for male (B) and female (C) mice. Statistical significance was determined using an ordinary one-way ANOVA with Dunnett's multiple comparisons test against the vehicle as calculated in Graphpad Prism 10.0. Percent inhibition is calculated from the mean of each group as compared to the vehicle and only statistically significant inhibition is shown (*** $p \leq 0.001$).

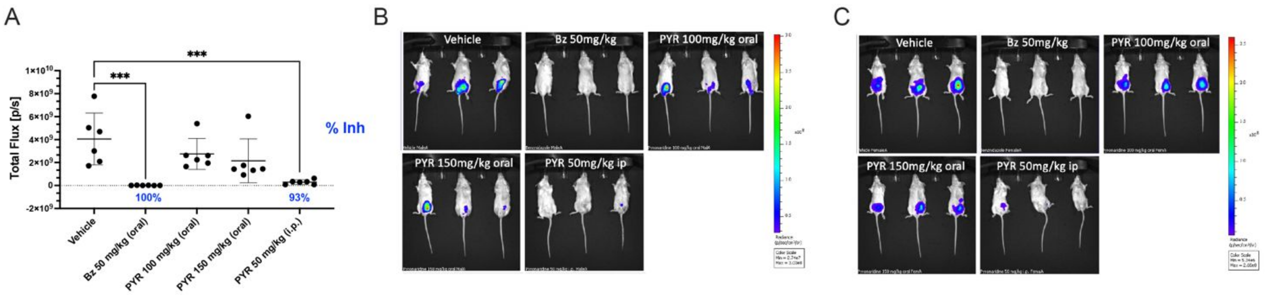

Figure S5. Plasma exposure of a single i.p. or p.o. dose (50 mg/kg) of pyronaridine in mice.

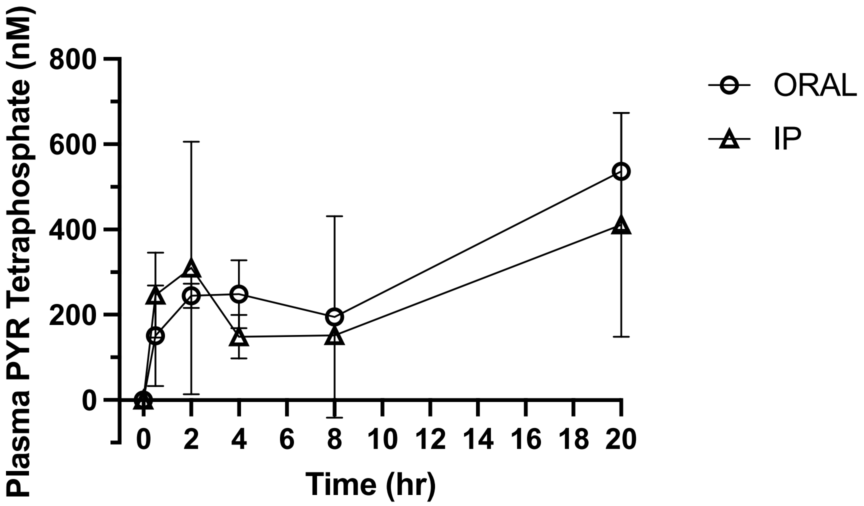

Figure S6. Assessing suboptimal antiparasitic dosing with benznidazole (Bz) activity against *T. cruzi* Br-luc 7 days post infection following treatment a four-day (days 3-6) treatment (oral). Comparison of total flux is represented graphically (A) based on the luminescent signal in mice following treatment with different concentration of Bz or vehicle (B). Statistical significance was determined using an ordinary one-way ANOVA with Dunnett's multiple comparisons test against the vehicle as calculated in Graphpad Prism 10.0. No statistically significant difference was seen from control, but % inhibition is calculated from the mean of each group as compared to the vehicle.

A

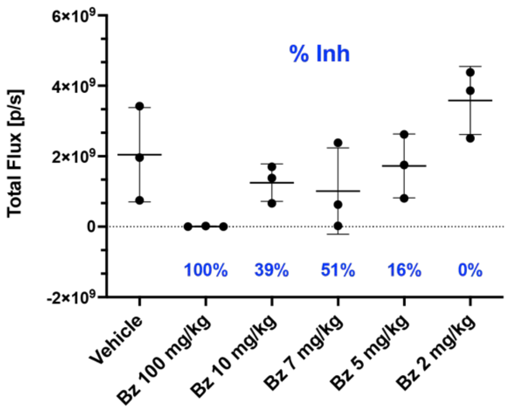

B

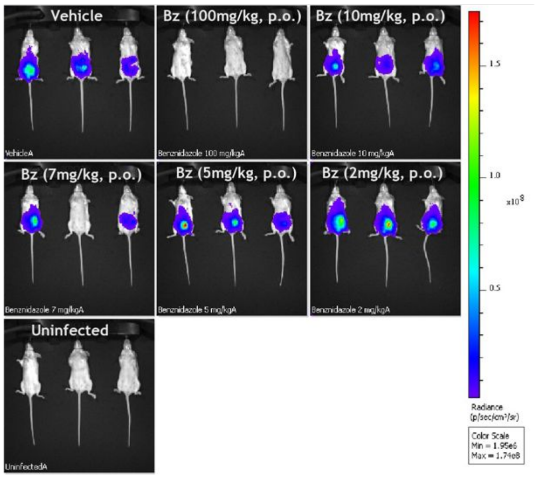

Figure S7. Mouse weights from chronic infection study. (A) Mouse weight, and (B) percent weight change. ‘Day 0’ is 3 months post infection. Both the pyronaridine (PYR) alone and PYR + Benznidazole (BZ) had statistically significant weight loss as compared to the uninfected vehicle control using an ordinary one-way ANOVA with Dunnett’s multiple comparisons test. analysis using Prism 10.0. (* p ≤ 0.05, ** p ≤ 0.01).

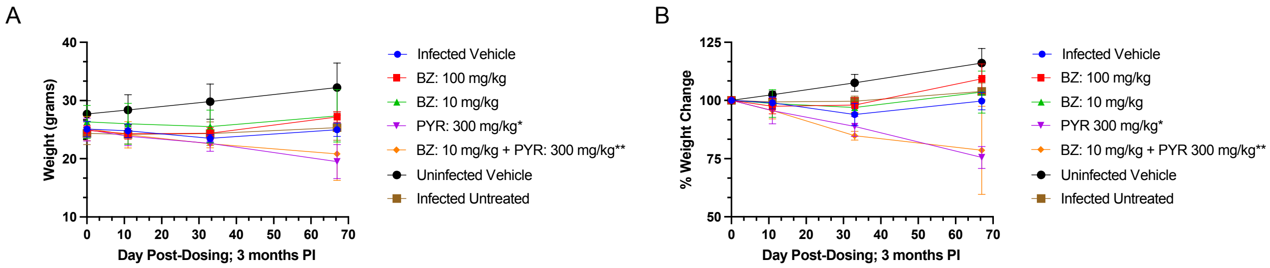

Figure S8. EKG Samples for chronic infection study 7- and 8-months post-infection (PI). Each pair (7/8 months PI) are representative examples from the same mouse. N/A signifies the EKG was unable to be taken.

Infected, BZ 10 mg/kg + PYR 300 mg/kg

7 Months PI

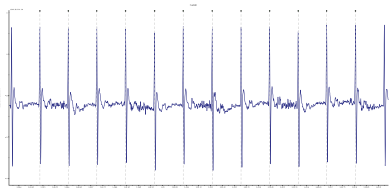

8 Months PI

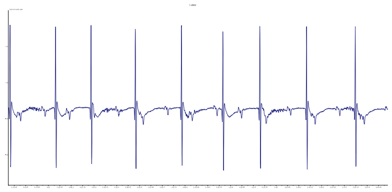

7 Months PI

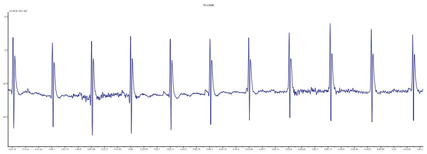

8 Months PI

N/A

7 Months PI

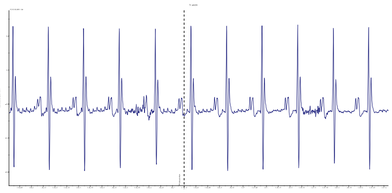

8 Months PI

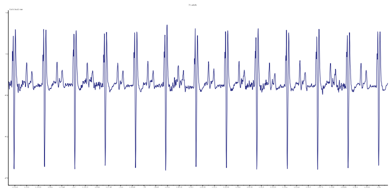

7 Months PI

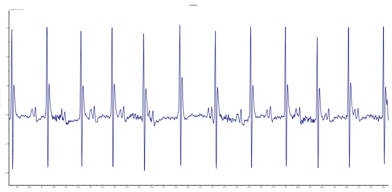

8 Months PI

N/A

Infected, BZ 100 mg/kg

7 Months PI

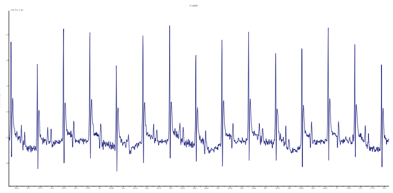

8 Months PI

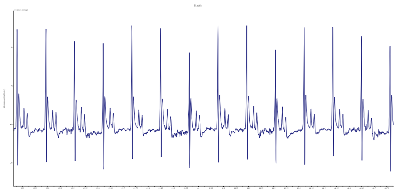

7 Months PI

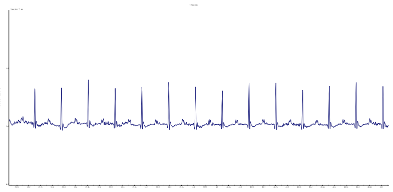

8 Months PI

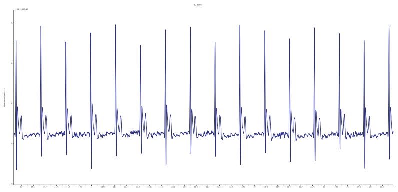

7 Months PI

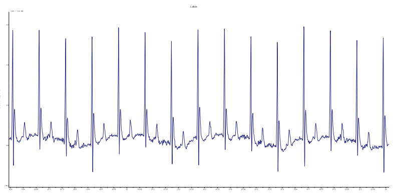

8 Months PI

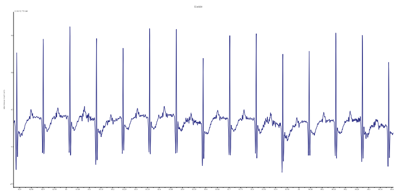

7 Months PI

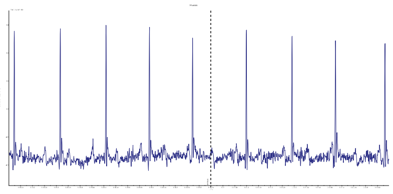

8 Months PI

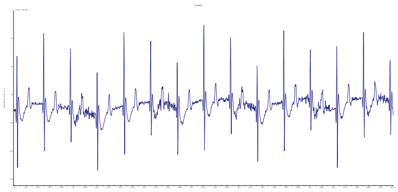

Infected, BZ 10 mg/kg

7 Months PI

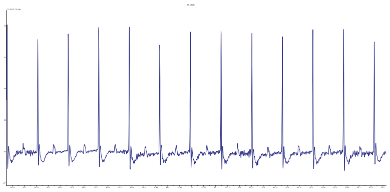

8 Months PI

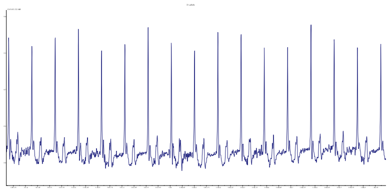

7 Months PI

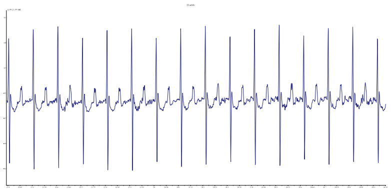

8 Months PI

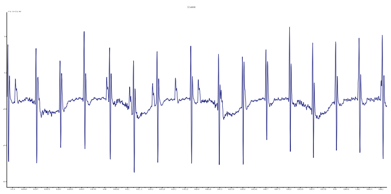

7 Months PI

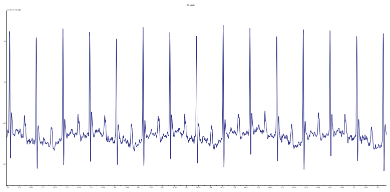

8 Months PI

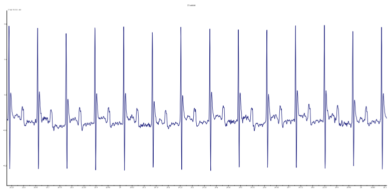

7 Months PI

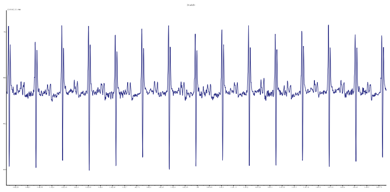

8 Months PI

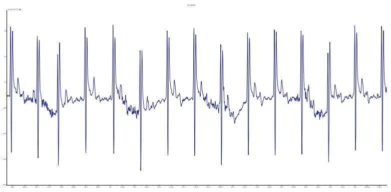

7 Months PI

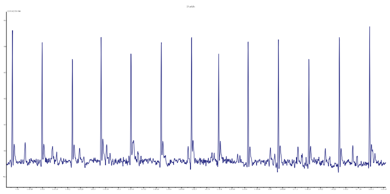

8 Months PI

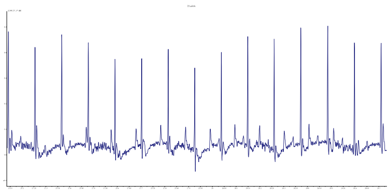

Infected, BZ 100 mg/kg

7 Months PI

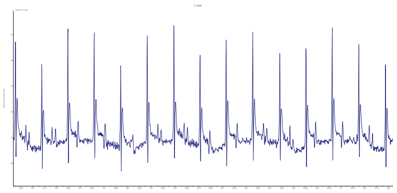

8 Months PI

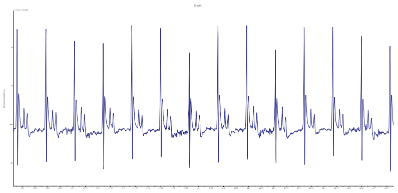

7 Months PI

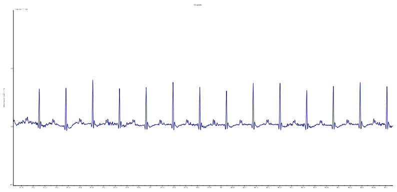

8 Months PI

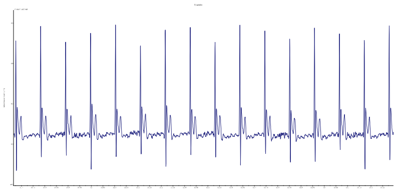

7 Months PI

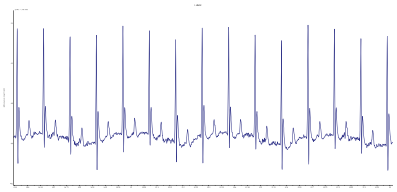

8 Months PI

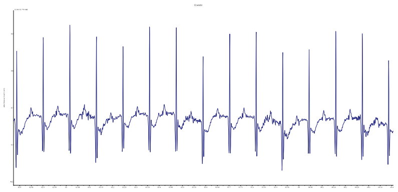

7 Months PI

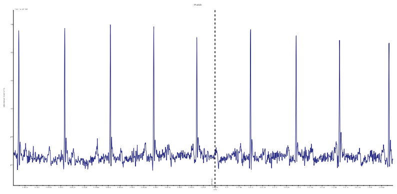

8 Months PI

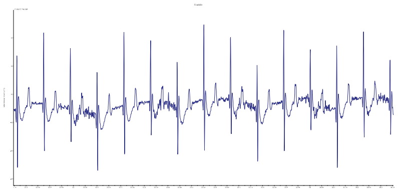

Infected, PYR 300 mg/kg

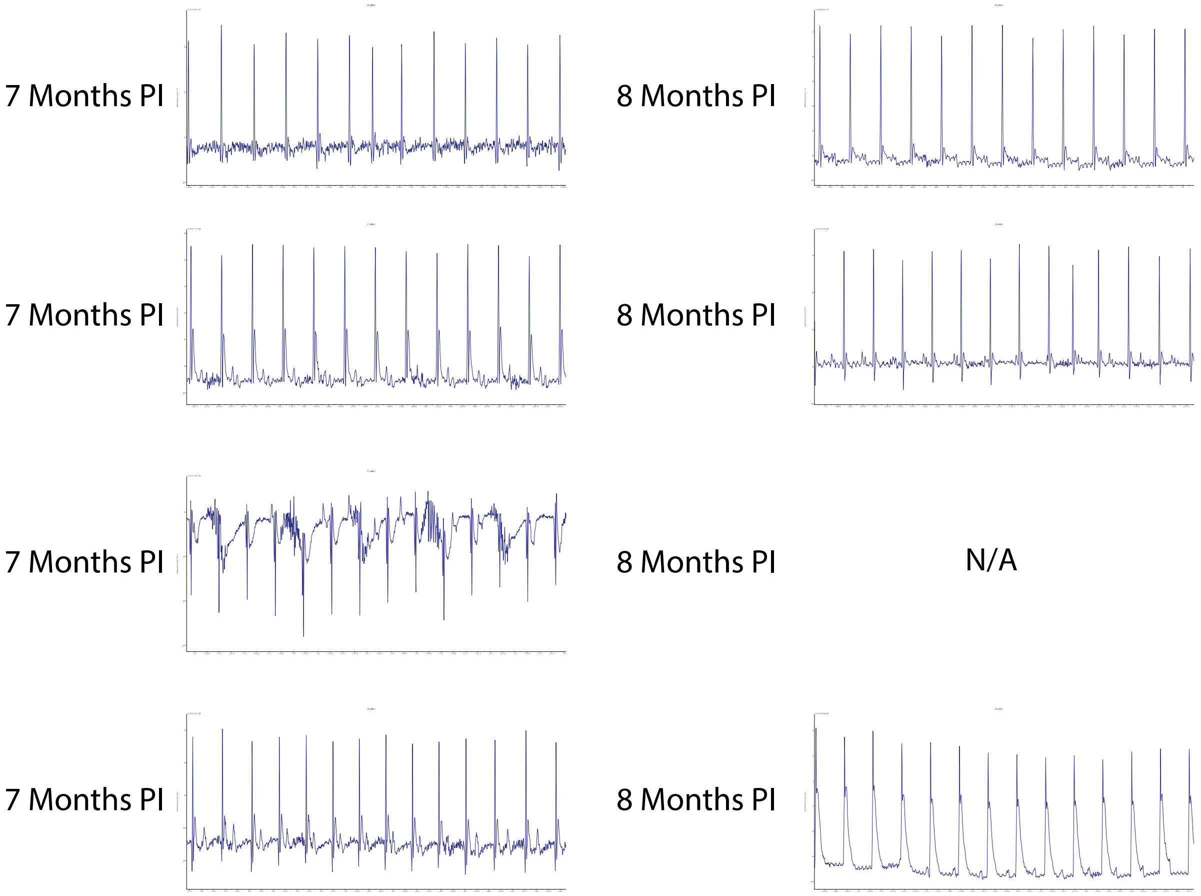

Infected, Untreated

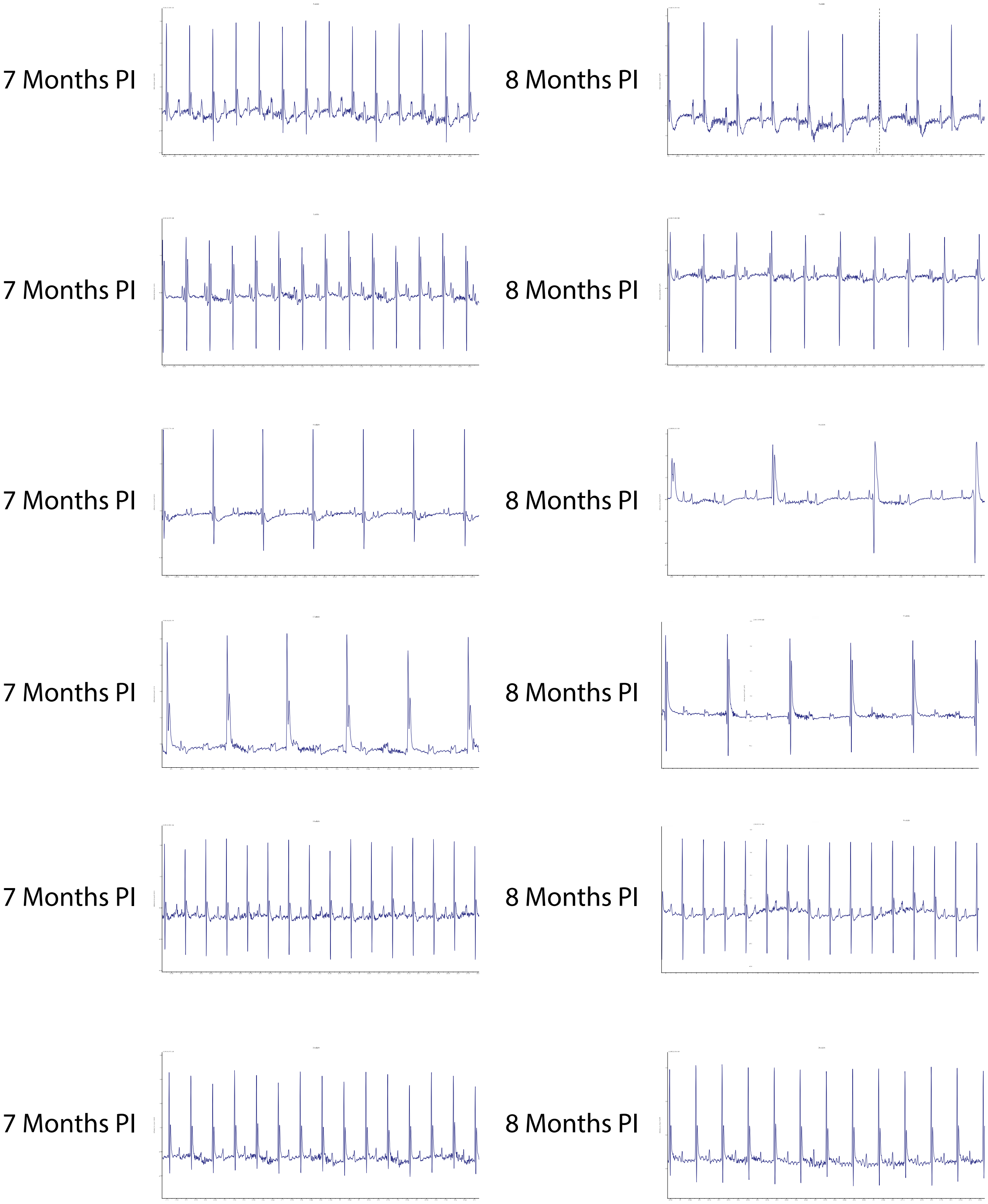

Infected, Vehicle Treated

7 Months PI

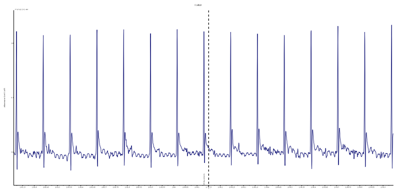

8 Months PI

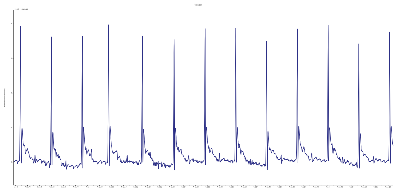

7 Months PI

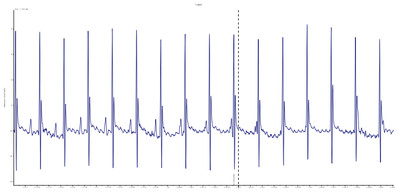

8 Months PI

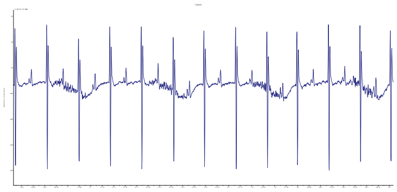

7 Months PI

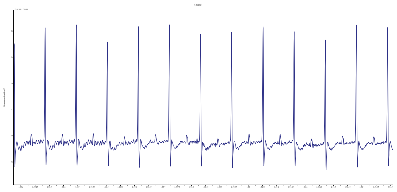

8 Months PI

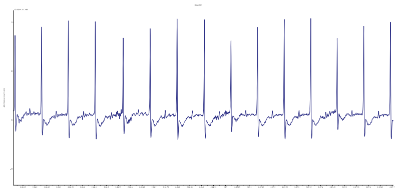

7 Months PI

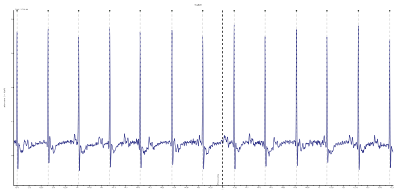

8 Months PI

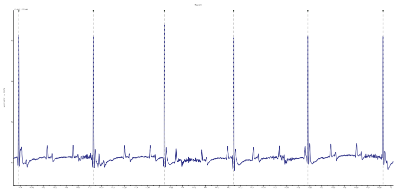

7 Months PI

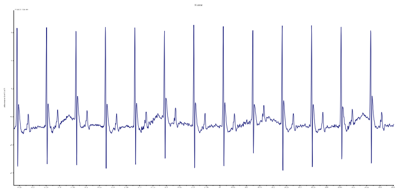

8 Months PI

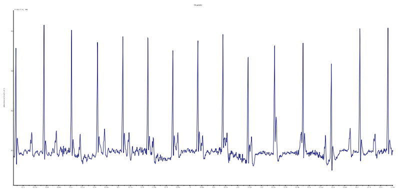

Figure S9. Results for from the chronic study against *T. cruzi* Sylvio X10/4. Parasite burden: (A) RT-PCR results from the collected heart samples. Statistical significance of the data (log transformed for RT-PCR) was determined using an ordinary one-way ANOVA with Dunnett's multiple comparisons test for each group as calculated in Graphpad Prism 10.0.2. (* $p \leq 0.05$, ** $p \leq 0.01$, *** $p \leq 0.001$, **** $p \leq 0.0001$). Bars represent SD from the mean (line). For RT-PCR, all values below the lower limit of detection (LLOD) are set to 0.5xLLOD.

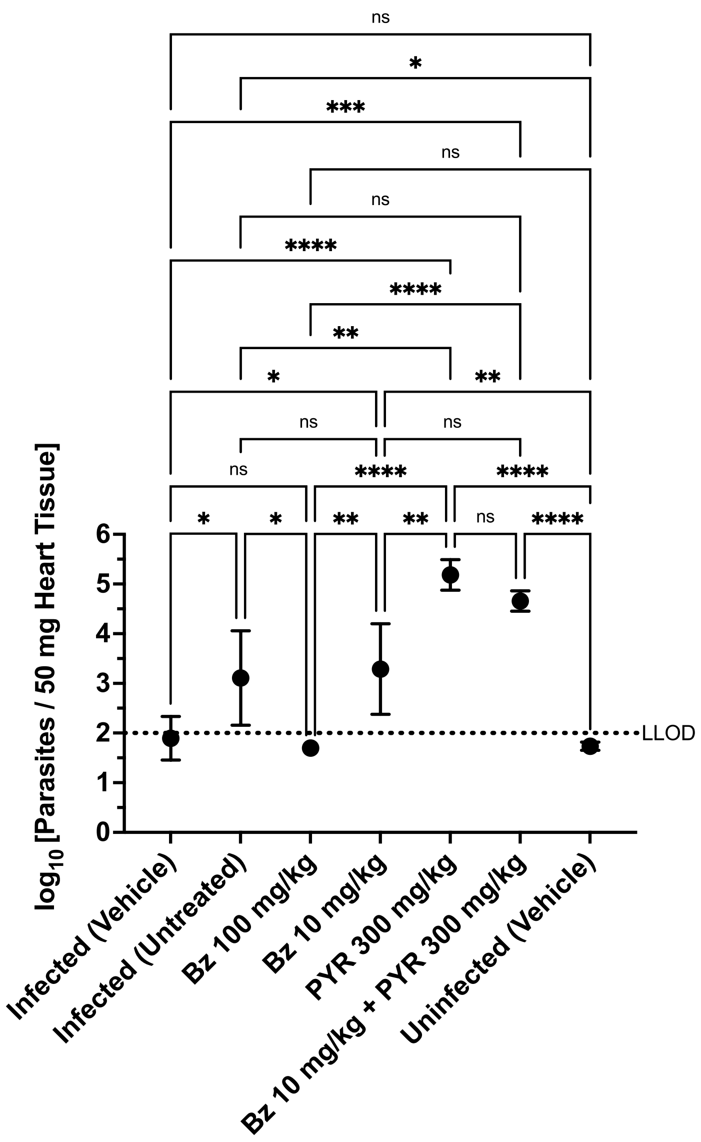
ns
*
***
ns
ns
****
****
**
*
**
ns
ns
ns
****
****
*
*
**
**
ns
****
log10[Parasites / 50 mg Heart Tissue]
0
1
2
3
4
5
6
LLOD
Infected (Vehicle)
Infected (Untreated)
Bz 100 mg/kg
Bz 10 mg/kg
PYR 300 mg/kg
Bz 10 mg/kg + PYR 300 mg/kg
Uninfected (Vehicle)

Table S1. Comparison of Pyronaridine efficacy in two different acute mouse models of Chagas disease. The *in vivo* study comparing the efficacy of different oral doses of pyronaridine against the CL-luc strain of *T. cruzi* to determine the minimum effective dose, did not result in the same levels of antiparasitic activity previously observed in the acute mouse model of Chagas disease infected with the Brazil-luc strain of *T. cruzi* and dosed by a different route (ip) (Ekins et al, 2015). A comparison table of the experiments is shown below:

	T. cruzi strain	Parasite load	Pyronaridine dose/ route	Vehicle	Parasite burden reduction after 4 days treatment
Ekins et al, 2015	Brazil-luc	10^6 parasites, ip	100mg/kg, bid, ip	Solutol 20%	83%
Current study, Dec/2017	CL-luc	10^3 parasites, ip	50, 100, 150mg/kg, po	Solutol 20%	<20%

ip: intraperitoneal; po: oral (gavage); bid: twice a day;

Table S2. Cardiac function assessment from mice in different time points post-infection and post-treatment. ND = not determined, N/A = not applicable, '?' represents ambiguity in the results.

Infection status	Treatment Group	Mouse #	3 Months PI	4 Months PI	5 Months PI	6 Months PI	7 Months PI	8 Months PI
Infected	Vehicle	6	Normal	Abnormal	Abnormal	Abnormal	Abnormal	Abnormal
Infected		7	Normal	Abnormal	Abnormal	Abnormal	Abnormal	Abnormal
Infected		8	Abnormal	N/A	Abnormal	Abnormal?	Abnormal?	Abnormal?
Infected		9	Abnormal	Abnormal	Abnormal	Abnormal	Abnormal	Abnormal
Infected		10	Normal	Normal	Abnormal	Abnormal	Abnormal	Abnormal
Infected	Benznidazole 10 mg/ kg	21	Normal	Normal	Abnormal	Abnormal	Abnormal	Abnormal
Infected		22	Normal	Normal	Abnormal	Abnormal	Abnormal	Abnormal
Infected		23	Normal	Abnormal	Abnormal	Abnormal	Abnormal	Abnormal
Infected		24	Normal	Abnormal	Abnormal	Abnormal	Abnormal	Abnormal
Infected		25	Abnormal	Abnormal	Abnormal	Abnormal	Abnormal	Abnormal
Infected	Benznidazole 100 mg/ kg	11	Abnormal	Abnormal	Abnormal	Abnormal	Abnormal	Abnormal
Infected		12	Normal	Abnormal	Abnormal	Abnormal	Abnormal	Abnormal?
Infected		13	Normal	Normal	Abnormal	Abnormal	Abnormal	Abnormal
Infected		14	Abnormal	Abnormal	Abnormal	Abnormal	Abnormal	Abnormal
Infected		1	Normal	Normal	Abnormal	No data	Abnormal	Abnormal
Infected	Pyronaridine 300 mg/kg	26	Normal	Abnormal	Abnormal	Abnormal	Abnormal	Abnormal
Infected		27	Abnormal	Abnormal	Abnormal	Abnormal	Abnormal	Abnormal
Infected		28	Abnormal	Abnormal	Abnormal	Abnormal	Abnormal	N/A
Infected		29	Normal	Normal	Abnormal	Abnormal	Abnormal	Abnormal
Infected		30	Abnormal	Abnormal	Abnormal	Abnormal	N/A	N/A
Infected	Benznidazole 10 mg/kg + Pyronaridine 300 mg/kg	31	Abnormal	Abnormal	Abnormal	Abnormal	Abnormal	Abnormal
Infected		32	Normal	Abnormal	Abnormal	Abnormal	N/A	N/A
Infected		33	Normal	Abnormal	Abnormal	Abnormal	N/A	N/A

Infected		34	Normal	Normal	Abnormal	Abnormal	Abnormal	N/A
Infected		3	Abnormal	Abnormal	Abnormal	Abnormal	Abnormal	Abnormal
Infected		4	Normal	Normal	Normal?	Normal?	Abnormal	Abnormal
Infected		5	Normal	Abnormal	Abnormal	Abnormal	Abnormal	Abnormal
Infected		16	Normal	Abnormal	Abnormal	Abnormal	Abnormal	Abnormal
Infected	Untreated	17	Abnormal	Abnormal	Abnormal	Abnormal	Abnormal	Abnormal
Infected		18	Normal	Abnormal	Abnormal	Abnormal	N/A	N/A
Infected		19	Normal	ND	Abnormal	Normal?	Abnormal	Abnormal
Infected		20	Abnormal	Normal	Abnormal	Abnormal	Abnormal	Abnormal
Uninfected		36	Normal	Normal	Normal	Normal	Normal	Normal
Uninfected		37	Normal	Normal	Normal	Normal	Normal	Normal
Uninfected	Vehicle	38	Normal	Normal	Normal	Normal	Normal	Normal
Uninfected		39	Normal	Normal	Normal	Normal	Normal	Normal
Uninfected		40	Normal	Normal	Normal	Abnormal	Abnormal	Normal
